# The role of total neoadjuvant therapy in locally advanced rectal cancer: a survey of specialists attending the All-Ireland Colorectal Cancer Conference 2022 including lead investigators of OPRA, PRODIGE-23 and RAPIDO

**DOI:** 10.1007/s11845-023-03591-4

**Published:** 2023-12-23

**Authors:** Timothy O’Brien, Geke Hospers, Thierry Conroy, Heinz-Josef Lenz, Jesse Joshua Smith, Emmet Andrews, Brian O’Neill, Gregory Leonard

**Affiliations:** 1 Patrick G Johnston Centre for Cancer Research, Queen’s University, Belfast, Northern Ireland; 2 Department of Medical Oncology, University Medical Center Groningen, University of Groningen, Groningen, Netherlands; 3 Medical Oncology Department, Institut de Cancérologie de Lorraine, Vandoeuvre-Lès-Nancy, Nancy, France; 4 Université de Lorraine, APEMAC, Équipe MICS, Nancy, France; 5 Division of Medical Oncology, Keck School of Medicine, Norris Comprehensive Cancer Center, University of Southern California, Los Angeles, CA USA; 6 Department of Surgery, Colorectal Service, Memorial Sloan Kettering, New York, NY USA; 7 Department of Surgery, Cork University Hospital, University College Cork, Cork, Ireland; 8 Department of Radiation Oncology, St Luke’s Radiation Oncology Network, Dublin, Ireland; 9 Department of Medical Oncology, University Hospital Galway, Newcastle Road, Galway, Ireland

**Keywords:** Rectal cancer, Total neoadjuvant therapy

## Abstract

**Background:**

The treatment of locally advanced rectal cancer (LARC) has evolved following recent landmark trials of total neoadjuvant therapy (TNT)—the delivery of preoperative chemotherapy sequenced with radiation.

**Aim:**

To assess the preferences of colorectal surgery (CRS), radiation oncology (RO) and medical oncology (MO) specialists attending the All-Ireland Colorectal Cancer Conference (AICCC) 2022 regarding the neoadjuvant management of LARC.

**Methods:**

A live electronic survey explored the preferred treatment approach and TNT regimen for early-, intermediate-, bad-, and advanced-risk categories of rectal cancer according to the European Society of Medical Oncology (ESMO) guidelines. The survey was preceded by an update from lead investigators of TNT trials (OPRA, PRODIGE-23 and RAPIDO), who then participated in a multidisciplinary panel discussion.

**Results:**

Ten CRS, 7 RO and 15 MO (32 of 45 specialists) participated fully in the survey resulting in a response rate of 71%. Ninety-four percent, 76% and 53% of specialists preferred a TNT approach for patients with advanced, bad, and intermediate-risk rectal cancer, respectively. A consolidation TNT regimen of long-course chemoradiotherapy followed by chemotherapy was the most preferred regimen. Upfront surgery was preferred by 77% for early-risk disease.

**Conclusion:**

This survey illustrated the general acceptance of TNT by rectal cancer specialists attending the AICCC as a valuable treatment strategy for higher-risk category LARC. Whilst the treatment of LARC changes, it remains best practice to individualize care, incorporating the selective use of TNT as discussed by an MDT and in keeping with the patient’s goals of care.

## Introduction

Neoadjuvant (chemo)radiotherapy followed by total mesorectal excision is a well-established standard of care for locally advanced rectal cancer (LARC), contributing to lower rates of local recurrence [1]. Despite this approach, almost a third of patients suffer distant metastatic relapse resulting in death in most cases [1, 2]. The impact of adjuvant chemotherapy in this context is unclear as many trials have suffered from poor accrual, low compliance and an inconsistent survival benefit leading to variable implementation across institutions [3–6].

The principal tenet of total neoadjuvant therapy (TNT) is that chemotherapy compliance improves when administered preoperatively in sequence with (chemo)radiotherapy, leading to a reduction in distant metastases. Additional benefits include the potential to downsize the primary tumour to improve surgical margins and provide a route to non-operative management (NOM) in selected patients with a complete clinical response.

Recently, multiple clinical trials, such as OPRA, PRODIGE-23 and RAPIDO, have demonstrated the benefits of TNT including an increased rate of clinical (cCR) and pathological complete response (pCR), improved disease-free survival (DFS) and the facilitation of NOM [7–9]. Although patient inclusion criteria and TNT regimens differed between the trials, the overall benefits are supported by a meta-analysis of eight studies totaling over 2000 patients [10]. As such, TNT is broadly recommended by the National Comprehensive Cancer Network (NCCN) for > T3 tumours, any node-positive disease or an involved or threatened circumferential margin [11]. On the other hand, the European Society of Medical Oncology (ESMO) rectal cancer guidelines predate the publication of many landmark TNT trials, and therefore, TNT does not feature prominently [12]. Furthermore, not all rectal cancer specialists are proponents of TNT given the lack of longer-term outcome data, especially overall survival (OS), as well as concerns of overtreatment [13, 14].

The primary goal of this survey therefore was to determine the TNT preferences of specialists attending the All-Ireland Colorectal Cancer Conference (AICCC) 2022, including the lead investigators of the influential OPRA, PRODIGE-23 and RAPIDO trials.

## Methods

**Fig. 1:**
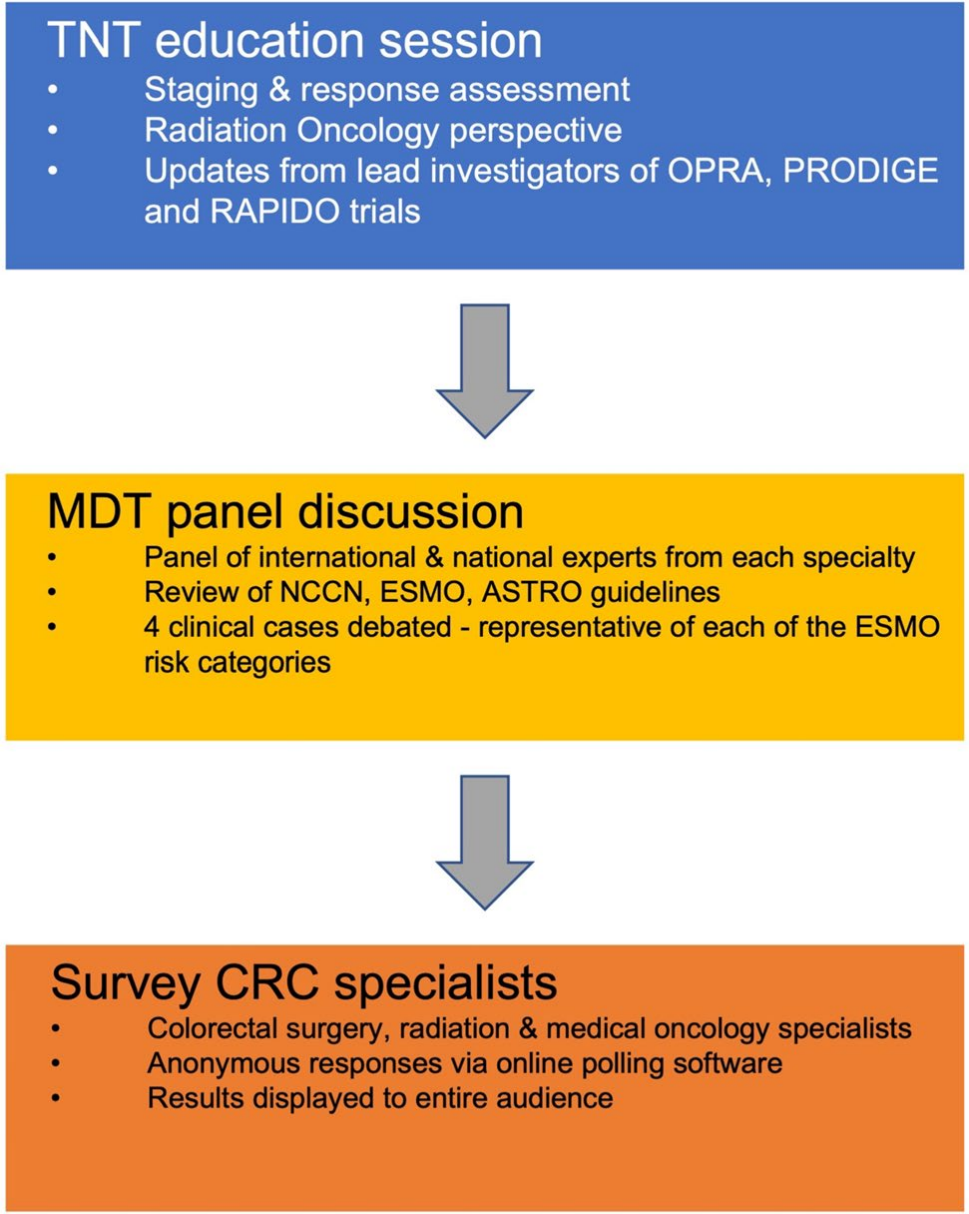
Overview of the survey methodology

We conducted a survey of colorectal surgery (CRS), radiation oncology (RO) and medical oncology (MO) specialists attending the national AICCC, an in-person-only event on 14 October 2022. An overview of the methodology is presented in Fig. 1.

Prior to the survey, conference attendees received a 90-min education session on rectal cancer featuring updates from lead investigators of OPRA, PRODIGE and RAPIDO. In the next session, these speakers formed an expert multidisciplinary panel consisting of 2 colorectal surgeons, 1 radiation oncologist, 3 medical oncologists and a radiologist. The current NCCN, ESMO and ASTRO guidelines were reviewed before 4 rectal cancer cases, including the history, radiology and histopathology that were presented to the panel in front of the live audience. The cases were formulated and agreed beforehand by the conference organizing committee, which consisted of a consultant in each specialty. The cases described stage II or III early (good), intermediate, bad, and advanced (ugly) risk categories of rectal cancer according to the ESMO clinical practice guidelines [12, 15, 16]. Stage I tumours were not discussed as TNT which has no role to play in this context.

**Fig. 2:**
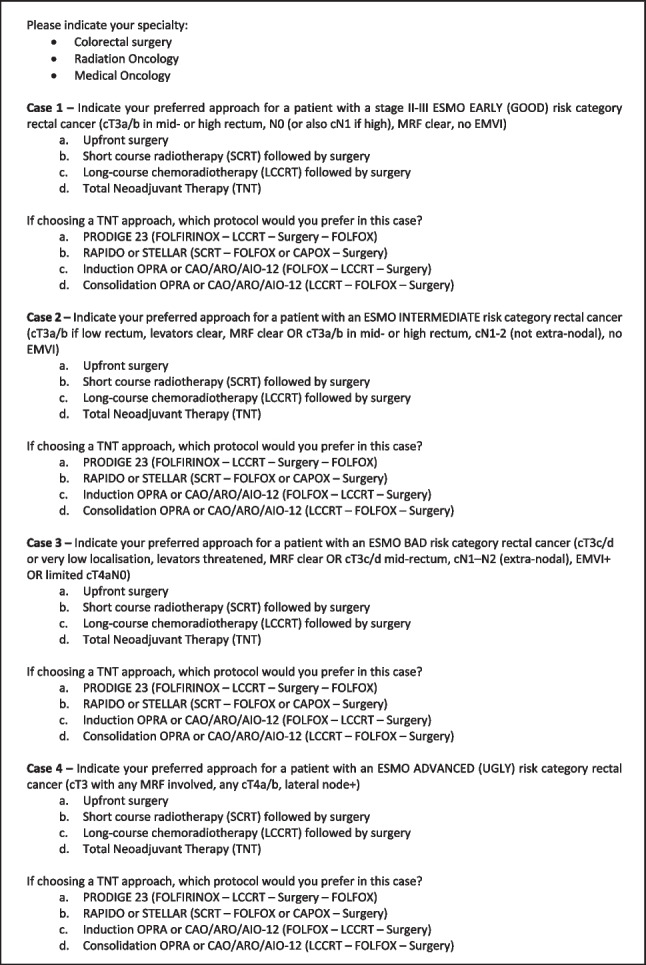
Survey questions

After the MDT panel had debated each case, all the CRS, RO and MO specialists attending the conference were invited to take part in a live, anonymous survey. Participants were informed that the survey was voluntary and were asked to provide their consent before proceeding. The survey consisted of four sections, each representing an ESMO rectal cancer risk category with TNM, extramural venous invasion (EMVI) and circumferential resection margin (CRM) descriptions provided. Each section asked the participant two multiple-choice questions: firstly, what was their preferred treatment approach for that category (upfront surgery, short-course radiotherapy (SCRT), long-course chemo-radiotherapy (LCCRT) or total neoadjuvant therapy (TNT)); secondly, if TNT was employed, what was their preferred regimen (PRODIGE 23, RAPIDO or STELLAR, induction OPRA or CAO/ARO/AIO-12, or consolidation OPRA or CAO/ARO/AIO-12). A description of the sequence of each regimen was provided. The survey is displayed in Fig. 2.

The survey was administered using online polling software (www.slido.com), accessed by the participant on their smartphone using a survey-specific code. Participants were familiarized with this software at the beginning of the conference with two practice questions. All participants were surveyed simultaneously with a maximum of 60 s allowed to respond to each question. Participants could only select one answer and undertake the survey once. The results of each question were subsequently displayed on the main conference screen to provide instant feedback to the audience.

Anonymized responses were downloaded into Microsoft Excel for coding. The rate of non-responses to each question was recorded. To improve validity, only respondents that confirmed their specialty were included in the final analysis. Descriptive statistics, chi-square/Fisher’s exact tests were performed using IBM SPSS statistics version 29. This survey is reported in accordance with the CROSS guidelines [17].

Please indicate your specialty:

- Colorectal surgery
- Radiation Oncology
- Medical Oncology

**Case 1 –** Indicate your preferred approach for a patient with a stage II-III ESMO EARLY (GOOD) risk category rectal cancer (cT3a/b in mid- or high rectum, N0 (or also cN1 if high), MRF clear, no EMVI).

- Upfront surgery
- Short course radiotherapy (SCRT) followed by surgery
- Long-course chemoradiotherapy (LCCRT) followed by surgery
- Total Neoadjuvant Therapy (TNT)

If choosing a TNT approach, which protocol would you prefer in this case?

- PRODIGE 23 (FOLFIRINOX – LCCRT – Surgery – FOLFOX)
- RAPIDO or STELLAR (SCRT – FOLFOX or CAPOX – Surgery)
- Induction OPRA or CAO/ARO/AIO-12 (FOLFOX – LCCRT – Surgery)
- Consolidation OPRA or CAO/ARO/AIO-12 (LCCRT – FOLFOX – Surgery)

**Case 2 –** Indicate your preferred approach for a patient with an ESMO INTERMEDIATE risk category rectal cancer (cT3a/b if low rectum, levators clear, MRF clear OR cT3a/b in mid- or high rectum, cN1-2 (not extra-nodal), no EMVI).

- Upfront surgery
- Short course radiotherapy (SCRT) followed by surgery
- Long-course chemoradiotherapy (LCCRT) followed by surgery
- Total Neoadjuvant Therapy (TNT)

If choosing a TNT approach, which protocol would you prefer in this case?

- PRODIGE 23 (FOLFIRINOX – LCCRT – Surgery – FOLFOX)
- RAPIDO or STELLAR (SCRT – FOLFOX or CAPOX – Surgery)
- Induction OPRA or CAO/ARO/AIO-12 (FOLFOX – LCCRT – Surgery)
- Consolidation OPRA or CAO/ARO/AIO-12 (LCCRT – FOLFOX – Surgery)

**Case 3 –** Indicate your preferred approach for a patient with an ESMO BAD risk category rectal cancer (cT3c/d or very low localisation, levators threatened, MRF clear OR cT3c/d mid-rectum, cN1–N2 (extra-nodal), EMVI + OR limited cT4aN0).

- Upfront surgery
- Short course radiotherapy (SCRT) followed by surgery
- Long-course chemoradiotherapy (LCCRT) followed by surgery
- Total Neoadjuvant Therapy (TNT)

If choosing a TNT approach, which protocol would you prefer in this case?

- PRODIGE 23 (FOLFIRINOX – LCCRT – Surgery – FOLFOX)
- RAPIDO or STELLAR (SCRT – FOLFOX or CAPOX – Surgery)
- Induction OPRA or CAO/ARO/AIO-12 (FOLFOX – LCCRT – Surgery)
- Consolidation OPRA or CAO/ARO/AIO-12 (LCCRT – FOLFOX – Surgery)

**Case 4 –** Indicate your preferred approach for a patient with an ESMO ADVANCED (UGLY) risk category rectal cancer (cT3 with any MRF involved, any cT4a/b, lateral node +).

- Upfront surgery
- Short course radiotherapy (SCRT) followed by surgery
- Long-course chemoradiotherapy (LCCRT) followed by surgery
- Total Neoadjuvant Therapy (TNT)

If choosing a TNT approach, which protocol would you prefer in this case?

- PRODIGE 23 (FOLFIRINOX – LCCRT – Surgery – FOLFOX)
- RAPIDO or STELLAR (SCRT – FOLFOX or CAPOX – Surgery)
- Induction OPRA or CAO/ARO/AIO-12 (FOLFOX – LCCRT – Surgery)
- Consolidation OPRA or CAO/ARO/AIO-12 (LCCRT – FOLFOX – Surgery)

## Results

One hundred and seventy delegates attended the AICCC including 17 colorectal surgeons (CRS), 7 radiation oncologists (RO) and 21 medical oncologists (MO). Forty-five participants answered at least one question in the survey. Thirteen were excluded from subsequent analysis as they did not specify their specialty. The response rate was therefore 71%. Data was 95% complete for this group.

### Early risk

**Fig. 3:**
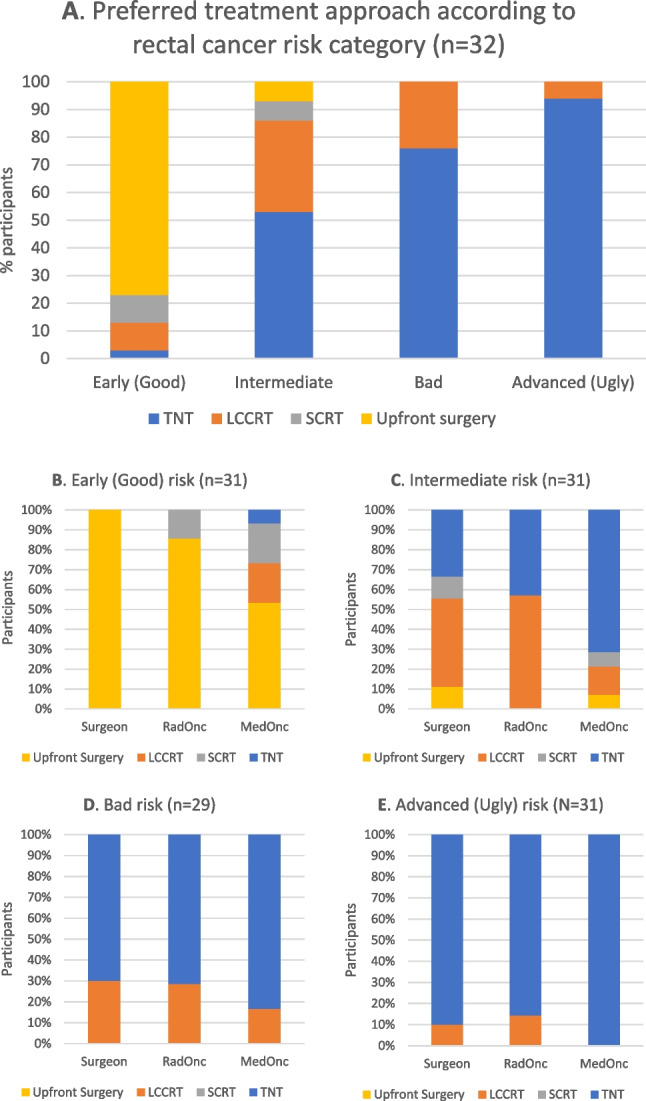
Preferred treatment approaches to ESMO rectal cancer risk categories, **A** all participants, **B**–**E** by specialist

Seventy-seven percent (24/31) of all specialists preferred an upfront surgery approach whilst neoadjuvant LCCRT and SCRT were preferred by 10% each (Fig. 3A). CRS and RO almost unanimously chose upfront surgery whereas MO were split between upfront surgery (50%) and a form of neoadjuvant radiation (40%) (*p* = 0.41) (Fig. 3B). Just one participant selected TNT for early-risk rectal cancer.

### Intermediate risk

The greatest heterogeneity of responses was observed in this category. Just over half of respondents (16/30) indicated a preference for TNT whilst a third chose LCCRT as the preferred neoadjuvant strategy. Seven percent of specialists opted for SCRT with another 7% preferring upfront surgery. TNT was selected most prominently by MO (71%) whilst LCCRT was most popular amongst RO (57%) and CRS (44%) (*p* = 0.30) (Fig. 3C).

**Fig. 4:**
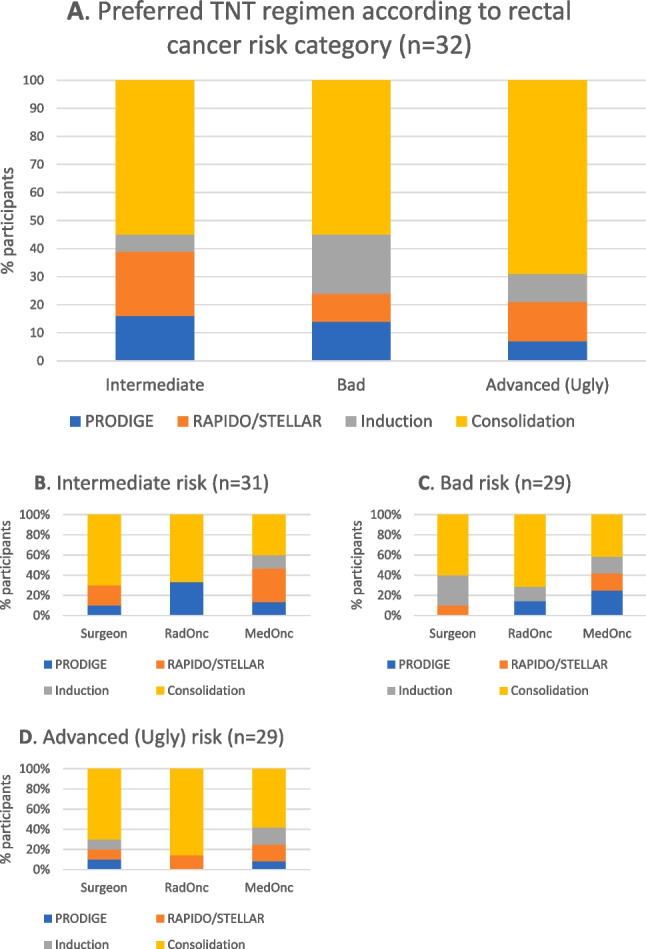
Preferred TNT regimens for each ESMO rectal cancer risk category, **A** all participants, **B**–**D** by specialist

If utilizing a TNT approach, 55% (17/31) selected a consolidation-type (OPRA or CAO/ARO/AIO-12) regimen, 23% a RAPIDO or STELLAR regimen, 16% PRODIGE-23 and 6% an induction-type (OPRA or CAO/ARO/AIO-12) regimen (Fig. 4A). The majority of CRS and RO preferred a consolidation regimen whilst MO were divided between consolidation (40%) and a RAPIDO or STELLAR regimen (33%) (*p* = 0.42) (Fig. 4B).

### Bad risk

The proportion of specialists preferring TNT increased for bad-risk rectal cancer as 76% (22/29) favoured TNT compared to 24% for LCCRT. There was no significant difference between CRS, RO and MO—at least 70% of each preferred TNT for this risk category (*p* = 0.75) (Fig. 3D). The most selected TNT paradigm was again a consolidation-type regimen, chosen by 55% (16/29). Twenty-one percent preferred an induction regimen, 14% PRODIGE-23 and 10% a RAPIDO or STELLAR regimen. The majority (> 60%) of CRS and RO preferred a consolidation TNT strategy whilst MO preferences were again divided across the TNT regimens (*p* = 0.60) (Fig. 4C).

### Advanced risk

There was almost an entire agreement amongst participants that TNT was the preferred approach for advanced-risk rectal cancer as selected by 94% (29/31) of specialists, with no significant difference between CRS, RO or MO (*p* = 0.29) (Fig. 3E). With regard to the preferred TNT sequence, 69% (20/29) opted for a consolidation-type regimen, 14% for a RAPIDO or STELLAR regimen, 10% induction and 7% PRODIGE-23. The majority (> 60%) of each specialty preferred a consolidation regimen (*p* = 0.98) (Fig. 4D).

## Discussion

The majority of specialists in this survey preferred a TNT strategy for advanced- and bad-risk category rectal cancers, which is supported by strong evidence from the major phase 3 TNT trials. Although TNT data has demonstrated improvements in complete response rates and DFS, our study clearly indicates that TNT is not mandated for all patients. In fact, the overwhelming majority of specialists preferred upfront surgery for early-risk, stage II and III rectal tumours. Meanwhile, the intermediate-risk category provoked the most heterogeneous responses from participants although approximately half opted for TNT. Overall, this survey showed that TNT is not a universal approach to LARC but rather a strategy that can be employed selectively to higher-risk patients on an individualized basis.

Across the intermediate, bad, and advanced-risk categories, a consolidation-type TNT regimen, such as that employed in the OPRA and CAO/ARO/AIO-12 trials, was consistently preferred in this survey. The consolidation arms of these trials demonstrated significantly higher rates of pCR (AIO-12) or cCR (OPRA) when compared to induction chemotherapy. In addition, 53% of patients in the consolidation arm of the OPRA trial avoided surgery at 3-year follow-up making this regimen attractive for those aiming for non-operative management (NOM), an approach that can improve quality of life by reducing low anterior resection syndrome (LARS). However, it must be noted that neither of these phase 2 trials significantly improved DFS or OS, and most centres do not recommend NOM unless as part of a clinical trial. Notably, at the American Society of Clinical Oncology (ASCO) 2023 conference, the 5-year OPRA update showed persistent differences in organ preservation using the consolidation approach (54% vs 39% with induction TNT) and lower rates of local regrowth (29% vs 44% with induction TNT). Furthermore, there was no oncologic detriment in either arm when integrating a watch-and-wait approach with salvage surgery for regrowth [18].

More than 60% of colorectal surgeons in this survey preferred a consolidation TNT approach, despite historical concerns about the potential risk of pelvic fibrosis as the time interval between radiation and surgery is extended. The French GRECCAR-6 trial showed greater surgical complications and morbidity when waiting for 11 weeks, as opposed to 7, after neoadjuvant chemoradiotherapy, which may partly explain the preference for the induction PRODIGE regimen in France. However, several trials have not demonstrated increased surgical difficulty or compromised R0 resection rates with a consolidation TNT approach [19–21].

Specialists in this survey demonstrated a low preference for the PRODIGE regimen, especially for advanced-risk disease for which it was chosen by less than 10%, surprising considering the robust data supporting this approach. PRODIGE-23, a phase 3, randomized controlled trial of induction FOLFIRINOX chemotherapy followed by LCCRT, demonstrated superior pCR rates (28% vs 12%, *p* < 0.0001), 3-year DFS (hazard ratio 0·69, *p* = 0·034) and metastasis-free survival (hazard ratio 0·64, *p* = 0·017) compared to LCCRT alone. Furthermore, since conducting our survey, additional follow-up data presented at ASCO 2023 demonstrated a significant increase in 5-year overall survival (6.9%), the only TNT trial to do so [22].

In the setting of an advanced-risk rectal cancer with inherent local symptomatology and metastatic threat, this triplet regimen aims to maximize primary tumour response whilst addressing micrometastatic disease early. However, there are some drawbacks to this approach. Firstly, adjuvant chemotherapy, which was mandated by the PRODIGE sponsors, remains controversial having failed to consistently show a survival benefit in rectal cancer. Secondly, there is the potential of overtreatment, particularly relevant considering the de-escalation of chemotherapy exemplified in node-positive colon cancer [23]. Thirdly, it is unclear whether the beneficial effects are due to the TNT approach or the addition of irinotecan. The precise value of adding irinotecan is being formally assessed in the JANUS phase 2 trial, which is comparing neoadjuvant FOLFIRINOX versus FOLFOX (NCT05610163). Interestingly this study was designed with significant input from colorectal cancer patient support groups that recommended a trial with more chemotherapy rather than more radiotherapy [24].

Short-course radiotherapy (SCRT) is widely considered to be equivalent to long-course chemoradiotherapy (LCCRT), yet the SCRT-containing regimens were preferred by a small number of specialists in this survey [25, 26]. RAPIDO, the largest phase 3 TNT trial, randomized patients to SCRT followed by chemotherapy, or standard neoadjuvant LCCRT [8]. The RAPIDO approach was originally deployed in patients with oligometastatic disease undergoing treatment with curative intent, the main advantage being the reduced time interval between radiation and systemic chemotherapy [27]. At the time of our survey, RAPIDO had demonstrated a marked improvement in pCR (28% vs 14%, *p* < 0.0001) and a reduction in distant metastases (20% vs 27% at 3 years, hazard ratio 0.69, *p* = 0.0048). However, a recent update has shown an increased risk of locoregional recurrence (10% vs 6%, *p* = 0.027) and no overall survival benefit compared to LCCRT, which has raised some questions. It is possible that a prolonged overall treatment time compared to LCCRT is deleterious to local control in a small subset of tumours that are poor responders to chemotherapy. To mitigate against this, a response evaluation with MRI and endoscopy could be incorporated mid-chemotherapy during induction or consolidation TNT approaches. Additionally, it is imperative to take into account the pre-treatment MRI to ensure a surgical approach that will optimize oncologic outcomes. In the RAPIDO study, which featured > 900 patients recruited from 54 centres, it was observed that downsizing occurred more often amongst the TNT group; however, fewer abdominoperineal resections (APRs) were performed in this group despite similar baseline tumour characteristics to the control arm. Finally, the STELLAR trial, which was similar to RAPIDO but conducted in a Chinese population, failed to improve DFS but improved OS, casting some doubt about the validity of their results [28].

Additional data published in the period since performing our survey reflects the continued evolution of this field as well as the increasing complexity facing clinicians.

The robust, mature survival data from the PRODIGE 23 trial may increase uptake of this induction chemotherapy approach, which the most recent NCCN guidelines recommend for > T3 tumours, any N + or locally unresectable disease [11]. However, the transitory decrement in quality of life by the addition of triplet chemotherapy must be considered [29]. In addition, it must be acknowledged that this regimen may be unfeasible in an older, frailer, real-world population, a cohort that has not been well represented in TNT studies to date [30].

Meanwhile, the PROSPECT trial, also presented at ASCO 2023, made the case for omitting radiation in lower-risk rectal tumours [31]. This randomized phase III trial compared standard neoadjuvant LCCRT vs neoadjuvant FOLFOX with omission of radiotherapy provided the tumour had regressed by at least 20%. Patients with T2N + , T3N − or T3N + tumours were recruited whilst those with more advanced diseases were excluded (distal, T4 tumours, threatened radial margins or > 4 lymph nodes). Similar rates of local recurrence, DFS and OS were reported, but importantly, radiation was omitted in over 90% of cases with neoadjuvant FOLFOX. This de-escalation approach might provide an alternative option in lower-risk patients keen to avoid radiotherapy.

Finally, neoadjuvant immunotherapy for patients with deficient mismatch repair protein (dMMR) colorectal cancer has gained further traction with data from a number of trials showing high response rates [28, 29]. In light of this new data and considering chemotherapy may be less effective in this molecular subgroup of tumours, NCCN has adopted immunotherapy as the preferred approach for dMMR/MSI-H rectal cancer with the option of non-operative management for those achieving a complete clinical response.

## Conclusion

This survey illustrated the general acceptance of TNT by rectal cancer specialists as a valuable treatment strategy for higher-risk category LARC. Most physicians agreed that early-stage tumours could be managed with surgery alone. Intermediate-risk cancer was deemed to require neoadjuvant therapy but provided the most heterogeneity regarding the optimal strategy. Most respondents preferred TNT for bad-risk cancer with almost complete unanimity for advanced-risk disease. There was a general preference for a consolidation TNT regimen in which neoadjuvant LCCRT is followed by systemic chemotherapy preoperatively. This approach may be favoured to facilitate a watch-and-wait, non-operative approach, which has gained increasing popularity amongst clinicians and patients alike. Finally, recent data demonstrating a survival advantage for induction FOLFIRINOX, the safe omission of radiotherapy for lower-risk cancers and immunotherapy for dMMR tumours highlight the need for an individualized treatment approach. It remains best practice to formulate a nuanced, bespoke treatment plan, incorporating selective use of TNT as discussed by a multidisciplinary team and in keeping with the patient’s goals of care.

## Data Availability

The survey data will be made available after publication upon request to the corresponding author.
